# Decolonial identities in the leadership coaching space: against neoliberal leader identity regulation

**DOI:** 10.3389/fpsyg.2024.1380610

**Published:** 2024-05-27

**Authors:** Sadi Seyama-Mokhaneli, Thato Belang

**Affiliations:** ^1^Department of Education Leadership and Management, University of Johannesburg, Johannesburg, South Africa; ^2^Transforma Business Solutions (Pty) Ltd, Johannesburg, South Africa

**Keywords:** leadership coaching, leader identity, Blackness, decoloniality, decolonial identity, leadership, black feminist pedagogy

## Abstract

The study uses the decolonial lens to disrupt the contentious dominance of whiteness in leadership development, not to mention in coaching, in management and organization studies (MOS). It contributes insights into how a decolonizing coaching space enables and guides a coachee to reflect and rethink the navigation of the realities of her decolonial identity. The decolonial identity encapsulates the authentic self and the neoliberal identity is the plastic self in a neoliberal university context. Universities' pervasive and normalized neoliberal discourse has become a “paradigm”—the overarching worldview through which universities' visions, missions, strategic objectives, and values are constructed. For academics to thrive in their performance and “walk on water” in achieving performance targets, they ought to embrace being academic capitalists, which shapes idealized neoliberal identities—conforming identities, complicit in undermining social, economic, and epistemic justice. Qualitative research methods were utilized to conduct a reflexive study, and data collected from the reflections and reflexive dialogues in leadership development coaching sessions and journals were thematically analyzed. The study reveals that the coach and coachee's shared decolonial identity offered counter-narratives that unmask the dominant great “white” man leadership in organizations. It also illuminates insights into the significance of black feminist pedagogy in the coaching process to honor the coachee's decolonial identity and rich cultural experiences. It enabled her to explore them critically and derive meanings from developing decolonizing, critically conscious leadership strategies for emerging transformation challenges. Meaningful dialogue dimensions emerged, which served as lenses that steered a decolonial approach in supporting the coachee to reflect and rethink the leadership performance vision, strategic objectives, action plans, implementation, and monitoring.

## 1 Introduction

In this article, we argue for the explicit expression of decolonial identities in the leadership development coaching space in neoliberally driven organizations to inspire the enactment of decolonial, critical-conscious leadership. Actioning a critical-conscious leadership would mean operationalizing a decolonized psyche (Seyama, 2024)—a subversion of the coloniality of power and being and an authentic commitment to the full humanity of black people [Black, Indigenous, and other people of color (BIPOC; Dei, 2017)]. Leader identity is crucial in leadership development because it undergirds people's cognition, behavior, emotions, and motivation (DeRue and Ashford, 2010). In this article, the coaching, undertaken within a women's leadership development program for university leadership, involved leader identity development and expression to enable the coachee's transition to a new leadership role. For the coachee, the decolonized identity represents the authentic self as a consciously black woman, flourishing in Blackness. The neoliberal identity represents the plastic self as the subjected identity serving neoliberal ideals (Ball, 2001) in a transforming context.

Organizations and universities in South Africa have been navigating political transformation since the end of apartheid. It has been an arduous and contested journey, exacerbated by the global neoliberal seduction of excellent, efficient, and effective organizations, which, if not critically considered, marginalizes the mandate of racial, economic, epistemic, and social justice. Myeza and April (2021) argue that black professionals in democratic South Africa's professional work environments experience racial, social, and economic marginalizations, inducing profound emotional distress, resentment, and overall emotional exhaustion. Similarly, Hlatshwayo (2020, p. 163) affirms black students and progressive black academics as “natives of nowhere,” confronted with the intersectionality of discriminatory, exclusionary, and epistemically violent postcolonial struggles. Ramohai (2019) established the intersectionality of gender and race in black academic women's experiences, undermining their humanity and professional progression.

Globally and nationally, the transformation of higher education aimed to expand access and opportunities for women to progress professionally and attain leadership positions. Nevertheless, Lipton (2018) cautions that the opportunities for women are primarily available to those open to adopting neoliberal principles and becoming neoliberal subjects performing within established managerialist structures. In the patriarchal dominance of gender inequalities, some women take up neoliberal subjectivities by shaping their neoliberal identities as academic capitalists and individualized competitors (Mavin and Yusupova, 2023). The neoliberal identities become conduits for achieving academics' neoliberal performances; they are notable and enabled and emerge as indisputable idealized identities. New leadership positions open opportunities for “integrating a leader identity with individuals' other valued identities” (Yip et al., 2020, p. 504). However, for some in new leadership positions, neoliberal identity conflicts with other existing valued identities (Karelaia and Guill'en, 2014). The neoliberal context that draws on the Euro-Western leadership paradigm has implications for how consciously black identities are enabled. Therefore, there are implications for coaching that while it is recognized “that a coachee's personal, professional and social identities are so implicitly bound up with aspects of the person and the ‘self”' (Jenkins, 2004), these identities could conflict.

In this article, as the coach and coachee, we were confronted with the coexistence of paradigmatically different decolonial (authentic) and neoliberal (plastic) polarized and incongruous identities. Hence, we draw on our reflections and reflexivities as the coach and coachee in responding to the question, “How is the coaching space enabling and guiding the coachee to reflect and rethink the navigation of the realities and complexities of her decolonial identity as the authentic self and neoliberal identity as the plastic self in a neoliberal university context?” In responding to the question, we also address the calls for decolonizing knowledge production in management and organizational scholarship (MOS; Banerjee, 2022).

In the next section, we first build a case against neoliberal identities in a transforming university context and reveal the coachee's contestation of the neoliberal “leader” label, which detracts from the spirit of decolonial identity. Second, we foreground the research on the decolonial theoretical lens. Third, we illuminate the black feminist pedagogy and its influence on the coaching approach where decolonial identities are expressed. Fourth, we provide a discussion of the reflexivity methodology as a tool that generated questions, insights, and rethinking that we captured in our reflection dialogues and journals. Fifth, we engage the meanings of a consciously black woman or Blackness identity as constructions of decolonial identity to ground the coachee's reflections and the coach's identity position. Sixth, we offer implications of the nexus of the coach and coachee's shared identities in the coaching space. We also provide an outline of the dimensions of the coaching dialogues that ground points of awareness, reflection, reflexivity, and rethinking that align the coachee's principles and aspirations with her authentic self while operating in spaces influenced by neoliberal norms. Finally, we conclude with significant emerging insights and future research recommendations.

## 2 Literature review

We briefly draw on literature to ground the coachee's problematization of the “leader label.”

### 2.1 The mainstream “leader label” as the perpetuation of neoliberal identity

The section provides the coachee's personal, contextual perspective, which underpins her contestation of neoliberal leader identities and related leader narratives in a transforming university context. Her position aligns with Raymond and Canham's (2022) observation that there are women in South African academia resisting racial patriarchy. In this sense, transformation signifies remedying the historical injustices (economic, social, and epistemic) faced by most black African people through exclusion and discrimination within the country's higher education sector (Breetzke and Hedding, 2018). This context grounds the coachee's realities and complexities of navigating leader development and coaching for a new leader role. For the coachee, a Blackness or consciously black woman's identity is valued, grounds her essence, and is inhabited as her authentic identity or authentic self. Nevertheless, such an identity is marginalized in mainstream leadership discourses. Thus, the coachee leader identity does not conform to a prevailing neoliberal narrative of conventional leadership (Nkomo et al., 2019).

This perspective ties in with her hesitation or caution toward participating in a business school's women's leadership program, including the allocation of a coach to guide her through the process of leader development. The challenge was being guided to draw on multiple identities' associated expertise, skills, and resources and utilize them in their respective roles (Creary et al., 2015, cited in Yip et al., 2020) while there is a lack of trust in the neoliberal identity. Oriented in learning and teaching leadership critically and identifying as a consciously black woman, the coachee was guarded against the colonizing epistemologies of business school programs.

Thus, alternative coaching identities and spaces were vital for her to guide her authentically and meaningfully in pursuing alternative leadership for transforming and decolonizing higher education toward inclusive diversity, accessible excellence, and future fitness. Having transcended the contradictions of identities and the anxiety of rejection for explicitly refusing the constructions of a black face in a white mask in the workplace, she firmly embraces the authentic decolonized identity. Thus, the coaching process required alternative thinking and pedagogy in exploring possibilities of drawing on decolonial identity to craft critical performatives or anti-neoliberal leading. Crucially, the coach's identity and positionality mattered. The worst experience for a black person is a coach or a development facilitator who approaches the process from a Blackness deficit perspective, assuming superiority over what the coachee needs to develop to accomplish the ideal leader title.

The coachee aligns with the arguments that the business school paradigm is complicit in culturing the neoliberal leader in MBA and executive leadership programs who creates colonial workplaces and pursues colonial capitalism (Dar et al., 2021). Moreover, it perpetuates racism and related microaggressions (Dar et al., 2021). Leadership theorization, practice, and teaching draw on the traditional Eurocentric-Western paradigm, which reinforces the great white man's leadership, privileging leader-centered approaches based on the reverence of whiteness. Various management and organization studies (MOS) have problematized whiteness scholarships that still set the tone of leadership epistemologies (Nkomo et al., 2019; Liu, 2022). Consequently, concerns about the racism of business schools are credible. It is crucial, therefore, to argue that there is limited criticality and decolonization in this space.

Sinclair (2007) points out that business school educational programs aim to develop students' expertise in technical knowledge without ingraining the necessary critical analysis of the outcomes and impact of that knowledge. Furthermore, the learning experience frequently resembled the negative aspects of corporate life, characterized by intense pressure, a hierarchical approach to knowledge, and organized interactions. Additionally, it mainly serves the agenda of furthering the influence and interests of an already advantaged elite (Learmonth and Morrell, 2019). Similarly, Collinson and Tourish (2015) critiques the conventional modes of learning facilitation that draw on the seductive transformational leadership approaches, heroizing the charismatic and visionary great white men. They argue that these hero-infused approaches disregard the fundamentals of unequal power dimensions in specific organizational settings and the possibilities of followers' opposition and rejection. Therefore, schooled in the conventional modes of thinking and doing leadership, the developed leader identities are of heroic leaders who hold dear the belief that power and agency should be vested in the hands of a few leaders (Collinson and Tourish, 2015). All others are followers who are expected to oblige and comply. However, in the relational leadership process, the leader and follower identities shift (DeRue and Ashford, 2010), with implications for disrupting superiority positionality.

The critique of neoliberalism in higher education is abound. Outside its discourse that strengthens capitalistic ideals and managerial approaches in universities, it has gained immense traction in shaping academics, administrators, managers, and leaders as neoliberal subjects (Seyama, 2022). This has implications for colonizing curricula, non-inclusive academe, and excluded students (Hlatshwayo, 2020). Academics' performances are neoliberally operationalized, culturing a colonizing instrumental engagement with teaching, research, and community engagement. For academics to thrive in their performance and “walk on water” in achieving performance targets (Seyama and Smith, 2013), they ought to embrace being academic capitalists, which shapes their neoliberal identities—conforming identities which risk complicity in undermining the authentic transformation mandate of equity, social, economic, and epistemic justice.

Of significance is that neoliberalism valorizes leadership as the ultimate organizational mode to empower organizations to transform and compete globally (Mavin and Yusupova, 2023). However, Learmonth and Morrell (2021) warn “that the language of ‘leadership' represents a particularly subtle but powerful opportunity for the pursuit of individual elite interests to be disguised so that it looks as if it is for the benefit of all” (p. 1). Universities' pervasive and normalized neoliberal discourses have become a “paradigm”—the overarching worldview through which universities' visions, missions, strategic objectives, and values are constructed.

Consequently, in academia, women are persuaded to adopt and navigate neoliberal subjectivities while confronted with gender discrimination and inequalities in universities. Mavin and Yusupova (2023) observe how women embracing individualized neoliberal competitiveness are, in fact, internalizing competition. In neoliberal settings, competition is one of the essential elements of leader identity, aligning with the leadership language of power, and women feel compelled to speak the right language.

As a coachee holding a positionality as a developing critical scholar, cautious of leadership's heroism, romanticism, and essentialism, which places assumptions of leader as great and followers as compliant subservient employees (Collinson, 2011), the self-declared “leader” label is problematic. I do not own the title of a leader. I cannot stand and declare myself a leader. I cannot assume the superioritized, elitist title normalized and presented as the apex of professional success. Instead, I opt for a leader social identity. According to DeRue and Ashford (2010), a leader's social identity denotes the “granting of that identity by relevant others” (p. 629). Learmonth and Morrell (2019) observe that the term “leader” “brings with it overwhelming pro-elite cultural associations—a set of assumptions that build up a picture of the social world that supports the interests of those in power” (p. 45). I have observed how neoliberal identities are polarizing and alienating those outside the power domain, positionally or socially (race, gender, class, sexual orientation). Therefore, as a coachee in the coaching space, I distance myself from the heroic leader or Me-dership (Learmonth and Morrell, 2019) of neoliberalism.

## 3 Decolonial theoretical lens

Decoloniality is conceived differently across local and global localities. In the context of this article, a decolonial lens is a positionality, perspective, interrogation, program, and practice of countering coloniality (Walsh and Mignolo, 2018). It critiques and offers insights into dominant colonial ideologies and discourses in shaping social, economic, political, racial, cultural, and epistemological power relations across different levels, including the global sphere (Banerjee, 2022). Intentional in its pursuit is the disruption and dislocation of the “colonial matrix of power” (Quijano, 2000, p. 550) and the reconstruction of emancipatory alternatives of our being, thinking, knowing, and doing (Quijano, 2007), separately and collectively. Mignolo and Walsh (2018) emphasize that:

It is intellectually, spiritually, emotionally, and existentially entangled and interwoven. The concern is with the ongoing processes and practices, pedagogies and paths, projects and propositions that build, cultivate, enable, and engender decoloniality; this is understood as a praxis—like walking, asking, reflecting, analyzing, theorizing, and actioning—in continuous movement, contention, relation, and formation (p. 19).

As an emancipatory endeavor, decoloniality refutes the reverence of European Enlightenment's rational project as the superior mode of being, thinking, and doing. It is a continuous process of unraveling colonialism in its varying dimensions, primarily its power dynamics of the oppressive social order of race, epistemology, gender, sexuality, spirituality, ecology, and languages and present-day forced labor (Grosfoguel, 2011 cited in Banerjee, 2022). In the postcolonial time, neoliberal subjectification is intentional and normalized. The neoliberal plot of strengthening colonizing capitalism and consumption is presented enticingly, deepening previously colonized people's inequalities and poverty (Mignolo, 2007). They are excluded from the profitable generational economic participation. Mignolo (2007) argues that “when people do not buy the package willingly or have other ideas of how the economy and society should be organized, they become subject to all kinds of direct and indirect violence” (p. 450).

In contemporary organizations, anti-black racism is prevalent (April, 2021; Sihela, 2022) and Mbembe (2016) contends that it is rooted and legitimized by colonial whiteness reinforced through apartheid practices. Thus, black identity in organizations is a dark cloud that triggers rejection, hostility, and injustice. At the identity level, decoloniality entails unlearning the colonized and inferiorized being and retracing and configuring the affirmed Blackness within individual and collective existence foregrounded by ancestral DNA and cultural heritage. The project is achievable through a paradigm shift and a conscientization of the coloniality of being. Quijano (2000 p. 534) notes that “race and racial identity were established as instruments of basic social classification,” and the colonized people's (BIPOC) enforced social identity was relegated to the bottom of the class ladder.

The decolonial lens foregrounds a quest for beings outside coloniality. It explores a decolonized identity—the authentic black African self, embracing and celebrating Blackness (Dei, 2017). Since the racial dimension stems from colonial foundations and personality and remains intact post-colonialism (Quijano, 2000), it is fitting that we continuously deconstruct and reconstruct contemporary black identities within the colonial matrix of power. The quest for the emancipation of black people is connected to adopting a decolonial standpoint, which involves speaking out and promoting the process of unlearning oppressive identities. Moreover, it also obliges drawing from the established and embraced decolonial identity to relate, think, create, and move through the existential spaces.

## 4 Black feminist pedagogy

Coaching is one of the diverse teaching methods in leadership programs (Collinson and Tourish, 2015). Thus, its pedagogy is vital because it influences how the coachees are orientated in developing leader identities and leadership approaches and strategies. Additionally, a coach as a learning facilitator drives the pedagogy within the coaching setting, and their positionality matters. The coach in this study, as a consciously black woman, aligning herself with discriminations, exclusions, and microaggressions of black women in organizations, foregrounds black feminist pedagogy in her work with coachees. Thus, in this reflection, the black feminist pedagogy served as a vehicle to meaningfully enable and guide the coachee's decolonial identity to center her leadership development. It fostered equitable teaching and learning (Mbulaheni et al., 2022). As the coach and coachee, we managed to engage in the dialectics of teaching and learning—learning from each other without issues of power asymmetries. Black feminist pedagogy is emancipatory and emanates from the intersectionality of black women's inferiorized and racialized beings, experiences of oppression and resistance (Crenshaw, 2017; Duncan, 2020). Operating within the business school localities, Dei (2017) reminds us that:

We live in an era where forms of education designed to win the consent of students, teachers, and the public to the inevitability of a neo-liberal, market-driven globalization process are being developed worldwide. In these hegemonic modes of pedagogy, questions about issues of race, class, gender, sexuality, colonialism, religion, and other social dynamics are simply not asked (p. ii).

Without question, for those in educational spaces, a complete dedication to decolonization requires them to establish environments where black people (BIPOC) can reestablish connections and actively participate in their knowledge systems and modes of understanding (Woods et al., 2022). Furthermore, within MOS, Cunliffe (2020) argues that against the tumultuous world that is characterized by inhumanity, environmental crises, and climate catastrophes, educators should conscientize their students and facilitate their reflexivity in becoming more critical and ethical in their leadership thinking, development, and practice. In this way, they can comprehend the problematizations of the dominant mainstream management and leadership and experience a paradigm shift. Black feminist pedagogy as a means to foster inclusive teaching and learning (Mbulaheni et al., 2022) is relevant in creating a decolonial coaching space. It guides learning approaches influenced by the historical struggles of black women against race, gender, and class discrimination (Mbulaheni et al., 2022). These strategies aim to embrace pluriversal and diverse epistemological perspectives. They also provide insights into patriarchal, hegemonic masculinist, racial, and class exclusions in learning spaces, curricula, and organizational settings.

Richardson (2018) posits that the black feminist pedagogy “keeps students and educators moving *forward* through anger and frustration, toward a praxis that acknowledges pain without becoming paralyzed by it” (p. 281). The traditional learning spaces demand students to cover up and maintain a prim and proper engagement with oppressive discourses, forcing them to suppress their pain and silence their voices. Black feminism counters this tendency by recognizing that black women's voices should be heard and authentically considered and that their voices cannot be complete unless they capture the lived experiences of being a woman within the characterizations of class, sexual orientation, and so on (Salane, 2018). It inspires learning settings that pursue anti-racism, anti-Blackness, and anti-sexism agendas (Salane, 2018). In acknowledging black women's voices as those of knowledge holders, black feminist pedagogy facilitates the quest to align with the calls for critical scholarship in MOS (Collinson and Tourish, 2015) and decolonization of MOS epistemologies (Banerjee, 2022). Consequently, the black feminist pedagogy facilitated meaningful reflections and reflexivities, resulting in meaningful insights with implications for the decoloniality of coaching spaces.

## 5 Methods

The research adopted reflexivity as a qualitative research approach to subjectively reveal the meaning-making of our decolonial identities during our coaching journey that signified a decolonial space. Reflexivity captures both self and critical reflexivity. Self-reflexivity connotes an interrogation of oneself's assumptions, attitudes, views, and ideals, and how we relate with others, our words, and actions toward them (Cunliffe, 2014). Critical reflexivity broadens the scope of interrogation to societal and organizational paradigms, historical and current power systematic structures, theories and epistemologies, and organizational regulations and practices (Cunliffe, 2014). The approach permitted us to be both participants and researchers in the women's leadership program. As Warwick (2011) argues, turning practitioners, managers, or students into researchers of their experiences and behavior offers discernments that are not accessible to an outsider researcher. Furthermore, outsider researcher's indirect attribution cannot fully explain the deeply political and impassioned power dynamics in people's direct interactions (Warwick, 2011).

Reflexivity fulfills the aims of critical research in unearthing different ways of knowing (Pillow, 2015). Critical research, “in its diversity, …shifts away from seeking knowledge for knowledge's sake toward embracing inquiry as a vehicle for connection, disruption, change, and resistance. In this vision, inquiry practices should reflect and further those justice aims” (Bailey, 2019, p. 93). We drew on Cunliffe's (2020) definition of reflexivity “as questioning taken-for-granted assumptions, practices, policies, and so on. [It] offers a way of developing more critical and responsible approaches to our intellectual strategies and to practical activities within the academic and corporate world” (p. 64). Our decolonial lens demanded that we transcend reflection—as “defined and practiced as a rational/thinking process where we employ logical reasoning to analyze a situation and/or ourselves with the aim of attaining a desired outcome” (Cunliffe, 2020, p. 65). Furthermore, this lens acknowledges the depth of the colonized minds of the previously colonized and colonizers, and our untenable lived experiences of discrimination, inequality, and injustice as black women in organizations. Cognizant of colonial power in organizations, reflexivity is a relevant tool to determine how our thinking and behavior could be inadvertently complicit in our oppression by embracing colonizing discourses (Fook, 2002). Therefore, our learning, thinking, and doing of leadership should be reflexive, that is, “questioning what we, and others, might be taking for granted—what is being said and not said—and examining the impact this has or might have” (Cunliffe, 2016, p. 741). And, importantly, as consciously black women embracing Blackness, reflexivity stands out because it affords measures to center our moral and ethical duty to both individuals and the world in which we live (Cunliffe, 2016, p. 741). Our critical perspectives on leadership thinking and practice should be foregrounded on equitable humanity, enabled by disrupting whiteness in organizational leadership. Engaging reflexivity as a developmental tool for the practice of leadership directed a critical interrogation of our coaching relationship within the women's leadership development program. We reflected and questioned dominant ideologies and values that underpin the organizational context, the aim of the program, and our mandates as coach and coachee in “living up” to the success of the program.

As co-authors, we engaged with the identity politics of our historical discrimination, which remains present through the coloniality of power. In revealing our journey, we confronted social and organizational power interwoven in the construction of our identities.

Our coaching sessions generated data by producing reflective and reflexive dialogues, which were recorded. They were conducted over several months between study blocks and post-completion of the program. Each session of the six was between 60 and 80 min and encapsulated the coachee's reflection, self and critical reflexivity on learnings in the program, and actioning her leadership within the organizational context. The coach's role was to interrogate the outcomes of the coachee's meaning-making processes and add value to assist her in accomplishing her responsibilities. Furthermore, the coach openly drew new perspectives from the coachee's critical scholarship of leadership and organizational studies. The self-reflexivity study did not require formal ethical clearance and written informed consent. However, we have taken proactive measures to ensure our research adheres to ethical principles and standards.

The coaching was the data generation space where the coach and coachee co-constructed the learning and unlearning of leadership development through reflection and reflexivity conversations. The captured learnings and unlearning are the data analyzed for this study. At the first level, for each session, the coachee had a draft of specific reflexive questions for the coachee, which were drafted continuously as informed by the previous sessions. From the responses to these questions, data emerged and was journaled. At the second level, the coachee brought a journal of reflections from the leadership development lectures and work encounters and would relate them. From the iterative conversations with the coach, further questions emerged to deepen the coachee's reflection on specific issues.

As participants and researchers, we continuously drew on the coaching sessions' reflective and reflexive dialogues to make sense of our impressions, ideas, questions, contestations, and shared knowledge. We used these to draft our written reflective journals that materialized our critical conversations. We thematically analyzed these to draw meaningful insights from the coaching journey that are presented in this article. We collaboratively undertook the manual coding process to construct themes. The first-order codes were built from the primary expressions of our identities and how we navigate these within white professional spaces. The second-order codes emerged through drawing from the decolonial lens. This way, we extracted codes that carried decolonial meaning-making of experiences, and learning and unlearning through the coaching journey. These were grouped into themes emerging as insights of explicitly expressing decolonial identities in the leadership development coaching space in neoliberally driven organizations to inspire the enactment of decolonial, critical-conscious leadership.

## 6 Findings and discussion

The findings and the discussion thereof capture the research's emergent merits of explicitly expressing decolonial identities in the leadership development coaching space in neoliberally driven organizations to inspire the enactment of decolonial, critical-conscious leadership. They are encapsulated in the main themes: Decolonial Identity: Owning Blackness and The Nexus of the Coach and Coachee's Shared Identities.

### 6.1 Decolonial identity: owning Blackness

This section reveals our expressions of decolonial identities as we reflected and reflexively interrogated our experiences, assumptions, and reactions during the coaching sessions. It explicates our meaning of decolonial identity and how sharing it established the atmosphere for connecting deeply as coach and coachee. It also delineates the issues the coachee comfortably divulged in the first meeting, establishing the groundwork for future critical discussions. Decolonial identity encapsulates what Dei (2017) refers to as a rearticulation of Blackness, Africanness, and black identity aimed at seeking a positionality that validates the challenging realities of the black and African experiences across different situations in the contemporary era. It fundamentally relates to an identity that resists the still dominant coloniality of whiteness identity. In the context of this article, decolonial identity is constructed as Blackness or consciously black woman identity as a response to Biko (2004) that black people should define themselves in their terms outside of white people while fighting for self-determination.

Biko (2004) declared, “as we proceed further toward the achievement of our goals, let us talk more about ourselves and our struggle and less about whites” (p. 50). This meant creating a “culture of self-assertion” (Mangcu, 2017, p. 285), taking the initiative to tell and act our own story about who we are, and countering fallacious, white-infused formation of the black character aimed at superiorizing the white character. As Mangcu (2017, p. 284) notes, Fanon and Biko aimed to inspire African's “actional racial moral identity” as opposed to “reactional identity.” Implications for leadership undergirded by a decolonial identity advance the decolonial project of recovering the full humanity of black people. At the same time, they foster decolonization as a continuous process of purging oppression of people—socially, culturally, economically, epistemologically, politically, and spiritually and restore their equitable humanity (Biko, 2004).

As the coach and coachee, we recognize ourselves as consciously black women who identify with Blackness as an affirmation of the black African race, encompassing our experiences, spirituality, aesthetics, culture, and languages. It was essential for me as a coachee to have this alignment within the coaching space. Identity work is an entry point for coaching in leadership development (Yip et al., 2020). It requires a deeply informed engagement, and to a large extent, the coach carries the key to unlocking the potential of marginalized decolonial identities.

#### 6.1.1 The coachee's expression of her identity position

Upon accepting the nomination to participate in the women's leadership program, I was conflicted about how to go along with the assumed dominance of the Euro-Western paradigm and whiteness epistemologies in business schools' teaching. I was more anxious about the coach—the extent to which I could authentically engage with my identity positionality and its influence on the meanings I make of organizational leadership, leaders, and followers. I have traveled a long journey of racism and discrimination as a black woman. I am at a point where I cannot assume a black inferiority position to conform to expectations and avoid tensions by ignoring the race elephant in the room and persistent coloniality in knowledge content and pedagogy and, alternatively, enabling the process to become a confessional of my inadequacies and seeking to strengthen my whiteness aptitude, thereby scaling up for my success as a leader. Given the opportunity to select a coach was a welcome reprieve. I felt a connection with the coach as a black woman, and I saw possibilities of declaring my identity without apology or sanitization. She represented a black woman who appeared and sounded like someone who owns her Blackness identity. Therefore, I felt safe from the nuanced shaming of my being—color, hair, aesthetics, culture, language, and accent.

During our first meeting, in response to the question—“Who are you?” I owned up to being foremost a spirit being before the material being that is largely socially constructed and chained in racialized and patriarchal biases. My essence emanating from my African spirit reveals, directs, clarifies, and shapes my awareness, understanding, and knowledge in ways the socialized material world cannot. Thus, I acknowledge myself as a knower of knowledge that falls outside the Eurocentric scientific knowledge. Fundamentally, I cannot logically account for some aspects of my work in pursuing my thinking and doing except to note that they are spiritually driven. It is imperative to proclaim the spiritual identity dimension within the coaching space because spirituality is hardly recognized in workplaces, especially in the diversity of African spirituality. Workplaces as professional environments are established on Eurocentric enlightenment rationality (Banerjee, 2022). Therefore, what is not scientifically intelligible has not had a legitimate space even though April (2021) argues that all three underpinning philosophical perspectives (sociological, psychological, and spiritual) that impact diversity, equity, and inclusion are equally imperative. Marginalized black professionals face further challenges when they express their African spirituality (Rapiya et al., 2023), for example, they experience anxiety, denunciation, depression, and weariness.

I explained how my identity forms the premise of my leadership thinking and practice. Also, obliged by my positionality to conceptualize decolonial thinking and practice in leadership, I could not place myself on the platter of the colonizing leader identity and leadership development. As a black woman, I do not take for granted the risk of regression to the unhealed apartheid wounds. With openness about my decolonial identity, I brought forward the hidden risks of owning Blackness within organizations in this era. Such people tend to be viewed suspiciously as radical troublemakers, and my observations and experience have shown how they are subtly excluded from strategic knowledge-sharing settings.

Furthermore, their legitimacy is questioned (Ramohai, 2019). Sometimes, their professional progression is interrupted (Mahlaula, 2019). Moreover, such settings worsen neoliberal surveillance and self-surveillance for those with “no power.” The advent of COVID-19 in increasing online work engagements in varying dimensions has enhanced surveillance capacities and people's anxieties about being under watch (Seyama, 2022). As a consciously black woman, I cannot be vulnerable in an unsafe space of dysconscious racism—racial ignorance, lack of knowledge, or sensitivity or what could be a judgment of my Blackness. I did not need to explain myself continuously with the shared decolonial identity within the coaching space. I was comfortable knowing that the coach would get the nuances of the pains of whiteness on a black body and soul and how these affect work engagement, performance, and wellbeing.

#### 6.1.2 The coach's expression of her identity position

At the core of my identity lies a commitment to critical and progressive thinking, a philosophy that champions open inquiry and continuous reevaluation of prevailing perspectives. This inclination to question extends beyond political ideology; it is a fundamental aspect of my engagement with the world. For me, critical questioning is a cognitive, emotive, and spiritual pathway to continuous personal and intellectual growth. Moreover, more significantly, it alerts one to the continuing risks of dominant ideologies' colonization of the mind in the post-truth era. Embedded within my identity is a profound connection to my African roots, a celebration of the diverse tapestry of cultures, traditions, and histories across the continent. It acknowledges the richness and heterogeneity within the term “African,” emphasizing the importance of amplifying marginalized voices and experiences often overshadowed in dominant narratives.

Navigating the coexistence of neoliberal and decolonial identities poses a unique challenge. While my critical reflexive perspective encourages adaptability and a dynamic approach to ideas, the decolonial identity calls for reevaluating entrenched racialized power structures and amplifying marginalized voices. Striking a delicate balance between the openness to change inherent in critical and progressive thinking and the critical examination of systemic issues embedded in decolonial perspectives becomes essential in this context. Rather than viewing neoliberal and decolonial identities as irreconcilable, I perceive an opportunity for synthesis toward critical performativity. The adaptability inherent in my critical inclination aligns with my commitment to inclusivity and equity, values informed by my unique black African identity. This synthesis is not a static achievement but a dynamic process, necessitating ongoing reflection, reflexivity, and adaptation.

Beyond these intersections, my identity is further enriched by a commitment to authenticity, transcending a mere personal attribute and ascending to a level of spirituality. Embracing authenticity involves aligning my thoughts, actions, and values with a profound sense of purpose and integrity. This spiritual dimension of authenticity underscores my commitment to being true to myself and contributing authentically to the discourse surrounding neoliberal and decolonial identities.

Laying out the coach's identity, positionality, and related role continues to unmask structures and systems of colonial power in organizational spaces. While we are of different generations, we established similar problematic experiences in contemporary organizations and delving into the coaching within our identities, we embraced what Dei (2016) posits as a necessary undertaking of decolonization that those with lived experience are knowers of knowledge and possess the right to voice it.

### 6.2 The nexus of the coach and coachee's shared identities

This section presents emerging insights from analyzing our reflective dialogues and journals from the coaching sessions. First, the section illuminates dimensions of our dialogues on how our declared identities inspired alternative agenda “items” for exploration, reflection, and rethinking. Second, it offers the coachee a place of decolonial insertion and coaching. Third, it aligns the coach's coaching with the coachee's decolonial identity positionality. The nexus of our shared identities as consciously black women represents how our commonalities got us to a mutual understanding of our shared perspectives and contestations of organizational leadership and leadership development standpoints. Fundamental to the nexus is how our alliances mediate against dominant mainstream leadership discourses, thus influencing a decolonial coaching relationship and process. This grounds the legitimization of black women as beings with voices in mainstream workspaces, stimulating emancipatory Blackness thinking and practices. As Dei (2016) notes, “our capacities to contest, communicate, and establish reference points and trajectories for the ideals of social justice, fairness, and equity; and our capacity to materially express these ideals make us human and are what restore Indigeneity” (p. 27).

#### 6.2.1 Dimensions of the dialogues

The nexus provides a more meaningful understanding of the impact of the expression of our decolonial identities to shape the how, what, and why of the reflexivity of our captured reflective dialogues and reflection journals. It reveals issues attended to in the coaching environment that straddle the dominant mainstream's contentious concerns. They represent the dynamics of critically reflexive coaching practice undergirded by decolonial identities. Engaging in the “subjective understandings of reality as a basis for thinking more critically about the impact of our assumptions, values, and actions on others” (Cunliffe, 2004, p. 407), the dimensions of the dialogues materialized. These are captured in Table 1.

**Table 1:** Shared identity dialogue dimensions.

1.	A painful awareness of the effects of imbalanced, gendered power relations in society filtering into organizations. Ramohai (2019) observed that black women's experiences of discrimination and microaggressions in institutions are founded on socio-historical racial patriarchal beliefs and values. Thus, it suggests using “negative experiences as a power base and springboard to build resilience and move forward toward successful transformation” (p. 1).
2.	The recognition of the intersectionality of black women leaders' struggles and drawing from them to deconstruct and reconstruct leader development in the coaching space. Thus, confronting the emancipatory battle from different points and shaping a holistic resistance position.
3.	The authentic acknowledgment that race and gender matter in the coaching space and should be explicitly and critically examined to position a setting that encourages a sense of psychological safety.
4.	Reiterating the argument that patriarchal socialization on gender impacts power culturing in organizations. Hence, women struggle to acquire, use, and maintain power.
5.	Challenging conventional thinking about how and when women should lead.
6.	Engagement with the rhetoric of transformation and decolonization in organizations and explicitly confront the organizations' shortcomings in considering the morality of neoliberal ideology.
7.	The influence of colonized and decolonized minds in society and organizations rearing its head in coaching spaces.
8.	Underscoring the conscientization of racial, gendered, professional hierarchies' power dynamics in coaching spaces.
9.	The imperative of coaching facilitated through the black feminist pedagogy. The black feminist pedagogy offers valuable insights and tools to leaders to create inclusive, empowering, and socially just organizational environments that recognize and value the diversity of individuals within the workplace. Ramohai (2019) contends, “this is only possible if black women understand their standpoints and locations and the influence that these might have on their perceptions” (p. 9).
10.	Barring the marginalization of those who critique normalized leadership thinking and practices. Moreover, problematize how this leadership foregrounds organizational structures, systems, and decision-making, continuing to serve colonial power.
11.	Rethinking leadership coaching from a critical leadership perspective and considering reflexive leadership. Leaders are not wondrous individuals holding supreme power, and followers are not people who unthinkingly submit to leaders' commands (Collinson and Tourish, 2015).
12.	Reconceptualizing leadership coaching as a co-constructed equitable, impactful, and meaningful transitioning that permeates common discernible positionalities and interdependent and complementary relations between the coach and coachee.
13.	Advocating for the emancipatory potential of both the coachees' and coaches' decolonial identities in the intentional emergence of the atypical black leader (Myeza and April, 2021), embracing Blackness.

Source: authors.

These dialogue dimensions are the lenses that guide a decolonial approach in supporting a coachee to reflect and rethink the leadership performance vision, strategic objectives, action plans, implementation, and monitoring. The points of thought, examination, and expression enabled us to set the agenda for our study (Jautz et al., 2023), offering assurance of respect and trust for the coachee and encouraging vulnerability in the settings. Highlighting these dimensions of reflection dialogues in the coaching environment can create an alternative coaching space for both the coach and the coachee. Furthermore, undertaken through reflexive engagement, they were vital in “developing a more collaborative, responsive, and ethical way of managing [and leading] organizations” (Cunliffe, 2016, p. 748).

#### 6.2.2 Location and conditions for decolonial insertion

As the coachee, first, the coaching dialogue dimensions enabled me to openly reveal leadership challenges and paradoxes in neoliberal contexts dominated by colonial power. Consequently, the coach could facilitate authentic critical reflection and reflexivity to guide me in seeing critical performativity opportunities, which are meaningful and effective performances that blunt the sharp edges of neoliberal performatives. Alvesson and Spicer (2012) refer to these critical performativities as anti-neoliberal performances, aiming to humanize workplaces. And to work through the expression of my sensitivities on the coloniality of mainstream discourses, openly declaring the legitimacy of decolonial insertions. Second, the decolonial coaching space permitted me to draw on my affirmed identity explicitly, and of importance was finding a place for it to boldly insert decoloniality and Blackness in leading with colleagues that still aligns with the organization's strategic objectives. It involved making “choices about conformity and deviance, appropriation and risk, isolation and friendship” (Cunliffe, 2018, p. 19). Third, the process strengthened leadership knowledge and methods to crafting emancipatory, inclusive, and socially just environments that embrace the worth of the diversity of individuals within the workplace.

These locations and conditions gave me opportunities to consciously and critically develop leadership strategic thrusts to initiate action in the noted organizational context.

By openly working through the noted organizational context, I suggest it creates prospects for self-crafting authentic power that would serve humane ideals while pursuing accessible excellence.

#### 6.2.3 Alignment of coaching with coachee's decolonial identity positionality

Our coaching journey has explored finding a meaningful place within my client's identity. This process has been about aligning her principles and aspirations with her authentic self while operating in spaces influenced by neoliberal norms.

##### 6.2.3.1 Affirming decolonial identity

Our coaching sessions have provided a space for my client to affirm and celebrate her Black consciousness. Through open dialogue and deep listening, we have unpacked her unique racial and cultural experiences and the significance of her identity within her leadership journey. By acknowledging and validating her heritage, we have laid a foundation for her to find authenticity and strength in her identity.

##### 6.2.3.2 Deepening decolonial identity

Coaching within this leadership development program aligns with the focus on identity (Nicholson and Carroll, 2013). However, it shifted from the predictable work of empowering the coachee to develop a new leader identity to deepen the existent decolonial for its flourishing in enacting alternative leadership.

##### 6.2.3.3 Challenging neoliberal norms

Our discussions have delved into the inherent contradictions between neoliberal values and my client's anti-neoliberal stance. By critically examining the influence of individualism, profit-driven motives, and hierarchical power dynamics, we have illuminated the spaces where her values intersect with systemic inequalities. This awareness has encouraged and empowered her to continue challenging these norms and carve out a space reflecting her decolonial convictions. And it was enabled through her acknowledgment of the organizational context illuminated in Table 2.

**Table 2:** Organizational context.

1.	Black women's limited share on the power table. They do not have adequate power bases, thus hindering their leadership.
2.	Black women remain on the peripheries of academe as knowledge producers.
3.	Eroded trust among people due to the organization's leadership and management's self-serving power bolsters prevailing power asymmetries and neoliberal individualism and competitiveness.
4.	People's constrained autonomy, low morale, and disengagement.
5.	Organizational leadership's hostile attitude toward people's critique of institutional decisions on the strategic direction.
6.	Individuals feel disenchanted with the authenticity of the organization's expressed transformation commitments.
7.	Continuation of inequitable practices because of persistent systemic barriers within the organization.
8.	Implicit marginalization of people within the organizational culture.

Source: authors.

##### 6.2.3.4 Empowering critical self-reflection and self-reflexivity

Our coaching journey has encouraged my client to engage in critical self-reflection and reflexivity, dissecting the layers of her identity and her roles in different contexts. Through exercises and thoughtful dialogue, we continue to explore the authentic and plastic selves, identifying the points of alignment and tension between these facets of identity. This process enables her to discern where she can authentically express her beliefs and where she might need to navigate the nuances of various roles.

##### 6.2.3.5 Reinforcing the reimagined leadership

We have deepened the trajectory of reimagining leadership beyond the confines of the “great man” archetype, which historically has centered on whiteness and perpetuated colonial ideologies. We outline a more inclusive and equitable leadership vision by weaving her Black Consciousness and decolonial values into her leadership approach. This approach encourages her to lead from a place of empathy, collaboration, and social responsibility.

##### 6.2.3.6 Developing strategies

Our coaching conversations are resulting in the development of practical strategies to navigate neoliberal contexts while remaining true to my client's decolonial identity. We continue to explore ways to communicate her values effectively, challenge systemic biases, and amplify her voice within spaces that may resist change. These strategies equip her to perform meaningfully while upholding her convictions.

##### 6.2.3.7 Powering emancipation

The coaching space has reinforced the coachee's extant emancipation praxis throughout our journey. By honoring her unique perspective and providing a platform for her to articulate her values clearly and boldly, we have facilitated a deeper understanding of her purpose and potential impact. This enablement has emboldened her to enact change within her spheres of influence while contributing to the broader decolonial movement.

Our coaching journey has been a transformative exploration of aligning my client's affirmed Black Consciousness, anti-neoliberal values, and skepticism toward traditional leadership with her identity. By navigating the complexities of her authentic self and plastic self within neoliberal contexts, we have created a space where she can authentically perform critically, inspire change, and contribute meaningfully to a more equitable and just world. This journey showcases the profound impact that coaching can have in facilitating personal growth, identity alignment, and social transformation.

We suggest that with these insights, we have started to contribute some answers to Dei's (2016), “How do we frame an inclusive, anti-racist, and anti-colonial global future, and what is the work that is required to collectively arrive at that future?” (p. 24).

## 7 Conclusion

The article is an outcome of an engagement with the question, “How is the coaching space enabling and guiding the coachee to navigate the realities and complexities of the decolonial identity as the authentic self and neoliberal identity as the plastic self in a neoliberal university context?” Thus, its crux is illuminating the emergent merits of expressing decolonial identities within the coaching space in business school's women leadership program. It addresses the need for more endeavors to interrogate self-identities in organization theory's research. The article puts forth the thesis that a coaching environment that dignifies the coachee's historically subordinated identity and endorses it as a laudable resource for rethinking leadership offers avenues for anti-elitist leader identity. It also fortifies learning and implementation efforts for decolonizing leadership in the quest for authentic transformation of organizations, including universities. In this context, the coaching process aligns with calls for decolonizing business schools and granting the coachee the opportunity to expand and deepen her pursuit of thinking and doing leadership differently. Uppermost to the coach's black feminist pedagogy was affirming the coachee's consciously black woman identity. The dialogues honored her identity and rich cultural experiences, enabling her to explore them critically and derive meanings from developing decolonizing, critically conscious leadership strategies for emerging transformation challenges.

The article also reveals how such a shared decolonial identity between the coach and coachee offers counter-narratives that debunk dominant whiteness ontologies and epistemologies as exclusively legitimized discourses in coaching for leadership development. Of significance is how the shared identities created a mutual foundation for interrogating the great man leadership, which reinforces coloniality in organizations. In this way, it expands the decolonial project in demonstrating that a coach's decolonial identity is part of the construction or meaning-making of the coachee's leadership development.

Additionally, the article unveils that while the deficit-oriented approach to coaching is dominant, with the coach as the knowledge carrier aiming to empower the coachee, there are possibilities of hidden insights in the untold, complex, and nuanced experiences of professional black women in their leadership journeys. Thus, the coach's critical self-reflection and reflexivity about the power imbalances in the coaching space were vitalized. Moreover, the coachee's critical self-reflection and reflexivity were pertinent in unmasking the wounds she experienced as a professional black woman in discriminatory workplaces. She was confronted by a realization that the emotive response to the quest of critiquing traditional leadership paradigms and offering alternative leadership risked conventionalists' subversion. Consequently, the thought-provoking conversations in the coaching space shed light on political intelligence or workplace politicking as a viable tactic to instigate critical-conscious leadership. Essential to politicking was building networks across the power continuum of those with and without power. Considering her position at the nexus of co-influencing in the transformation context, she critically reflected on showing up authentically and purposefully and thinking through opportune moments to ask the difficult questions, acknowledge transformational progress, highlight shortcomings, and prompt thoughts on tackling the deficiencies. In doing this, the coachee was powered to find a place to perform meaningfully within her decolonial identity as an authentic self.

There is immense potential in drawing on decolonial identities to disrupt the coloniality of leader identity and leadership development within coaching spaces. Future research could be valuable in exploring coaching relationships where decolonial identity is shared more widely across races and genders. Furthermore, the research could interrogate how the coach's declared decolonial identity could influence coachees who hold the dominant Eurocentric or conventional leadership perspectives. We acknowledge the risks related to this, particularly the reported dismissiveness, anger, and discomfort of such participants. However, such a study is imperative amidst the evident failing global traditional great man's leadership. Its deposition is urgent if we are to save the world.

## Data availability statement

The original contributions presented in the study are included in the article/supplementary material, further inquiries can be directed to the corresponding author.

## Ethics statement

Ethical review and approval was not required for the study on human participants in accordance with the local legislation and institutional requirements. Written informed consent from the patients/participants was completed and signed to participate in this study in accordance with the national legislation and the institutional requirements.

## Author contributions

SS-M: Conceptualization, Data curation, Formal analysis, Investigation, Methodology, Validation, Visualization, Writing – original draft, Writing – review & editing. TB: Conceptualization, Data curation, Formal analysis, Investigation, Project administration, Validation, Writing – original draft.
